# TROCHANTERIC FRACTURES: ARE THE CLASSIFICATIONS RELIABLE?

**DOI:** 10.1590/1413-785220243201e279781

**Published:** 2025-04-07

**Authors:** LUIS HENRIQUE ZAMBRA WIN, OLIVER DAMIANI MEYER, EDUARDO PEDRINI CRUZ, IVAN SIMIONATO, CARLOS ROBERTO SCHWARTSMANN, FERNANDA COUTINHO KUBASKI

**Affiliations:** 1 Santa Casa de Misericórdia Medical Center, Porto Alegre, RS, Brazil.

**Keywords:** Trochanteric fracture, Classification, Reliability, Interobserver agreement, Fratura Trocantérica, Classificação, Confiabilidade, Concordância Interobservadores

## Abstract

**Objective:**

To evaluate classification use based on the assessment of agreement in trochanteric fractures between different observers through a simple proposal of bimodal classification.

**Methodology:**

A total of 50 radiographic images of femur trochanteric fractures were selected and classified by 22 evaluators, 10 traumatologists and 12 residents, as STABLE or UNSTABLE. The assessment of reproducibility was done using the kappa statistical index. After the evaluation, the groups were isolated and submitted to the ANOVA test with Bonferroni correction to evaluate the statistical differences between them.

**Results:**

When evaluated by the kappa index, the results of the 50 fractures assessed by the 22 evaluators were k = 0.272. The reproducibility of the classification proposal was considered statistically weak.

**Conclusion:**

Based on this study, it is recommended that the classifications be extensively tested and reach a minimum level of interobserver reproducibility (kappa > 0.8). It is suggested that classifications that do not achieve this result be improved or abandoned. ***Level of evidence II, Prospective study.***

## RESUMO

**Objetivo:**

Avaliar a utilização das classificações a partir da avaliação de concordância nas fraturas trocantéricas entre diferentes observadores através de uma proposta simples de classificação bimodal.

**Métodos:**

Foram selecionadas 50 imagens radiográficas de fraturas trocantéricas do fêmur e classificadas por 22 avaliadores, sendo 10 traumatologistas e 12 residentes que definiram as fraturas como ESTÁVEIS ou INSTÁVEIS. A avaliação da reprodutibilidade foi dada pelo índice estatístico Kappa. Após a avaliação, os grupos foram isolados e submetidos ao teste ANOVA com correção Bonferroni para avaliar as diferenças estatísticas entre eles.

**Resultados:**

Quando submetidos a avaliação pelo índice Kappa os resultados das 50 fraturas avaliadas pelos 22 avaliadores foi de k = 0,272. A reprodutibilidade da proposta de classificação foi considerada estatisticamente fraca.

**Conclusão:**

A partir deste estudo recomenda-se que as classificações sejam extensamente testadas e atinjam um nível mínimo de reprodutibilidade (Kappa > 0,8) interobservadores. Sugere-se que as classificações que não atinjam este resultado sejam aprimoradas ou abandonadas. **Nível de evidencia II, Estudo prospectivo.**

## INTRODUCTION

Classifications are a part of orthopedists’ routines since their training. Based on them, it is possible to classify fractures, degrees of arthrosis, and other injuries, facilitating the diagnosis, stratification, treatment, and prognosis. It is assumed that classifications are an important tool in our arsenal and that they are used homogeneously by all evaluators, therefore, being useful in daily practice. Does this really happen?

In the literature, there is a vast number of studies verifying the interobserver agreement of orthopedic classifications. In this study, we isolated those that treat trochanteric fractures and evaluated the reproducibility of a fictitious classification for trochanteric fractures, which is extremely simplified: STABLE and UNSTABLE. 

Like in other studies, we used the kappa index or coefficient, first presented in 1960 by Cohen, as an indicator of reliability; this index is a way to evaluate intra and interobserver agreement by adjusting for random agreement. The index of agreement between observers is measured using a scale from -1 to 1. The closer the index gets to one, the greater the agreement, and the lower the number, the lower the agreement.^1^ ^)-(^ ^4^

The objective of this study is to evaluate the interobserver agreement of 50 radiographic images of trochanteric fractures among 22 traumatologist and resident observers.

## MATERIALS AND METHODOLOGY

**Figure 1:**
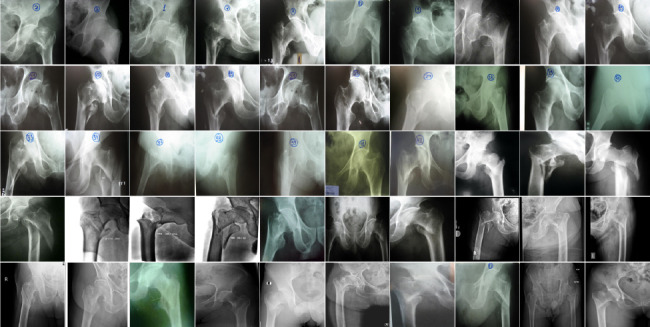
Image of the 50 radiographs used for the assessment.

A total of 50 radiographic images of femur trochanteric fractures without patient identification were randomly selected from our residence database (Figure 1). All radiographies were anteroposterior (AP), performed without traction prior to the reduction maneuver.

The radiographies were evaluated by 22 observers, 10 traumatologists and 12 residents (three 3^rd^ year, four 2^nd^ year, and four of 1^st^ year students). An explanation about fracture classification was previously carried out, and the observers were asked to classify one fracture as STABLE or UNSTABLE. A stable fracture was one in which the muscular forces involved in the focus keep the fragments in the anatomical position, without secondary deviations. Unstable fractures are those in which the muscle forces involved in the fracture focus tend to displace the fragments. 

The images were individually projected and numbered in a sequence from 1 to 50. Each image was projected for 30 seconds, and the protocol was completed individually by each observer.

The data were analyzed and interpreted by a statistician using the kappa agreement test. Subsequently, the results were analyzed using the ANOVA variance analysis with Bonferroni correction.

## RESULTS

Results were grouped into 4 sets according to the evaluator’s professional experience. Traumatologists were in the first group, last-year residents were in the second group, the rest of the residents were in the third group, and the fourth group was comprised of all observers.

**Graph 1:**
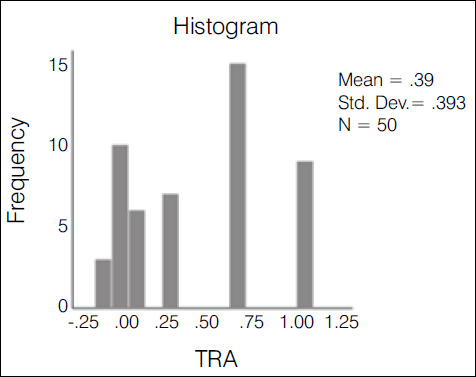
Histogram of the variation and concentration of kappa indices among traumatologists

**Graph 2:**
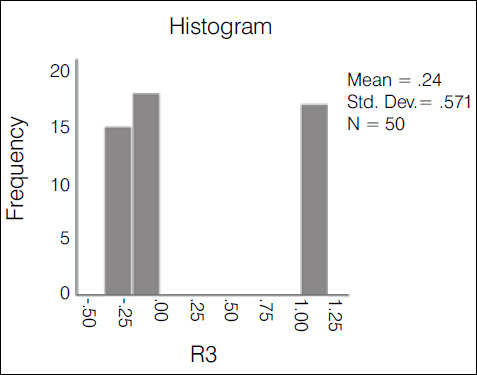
Histogram of the variation and concentration of kappa indices among R3

**Graph 3:**
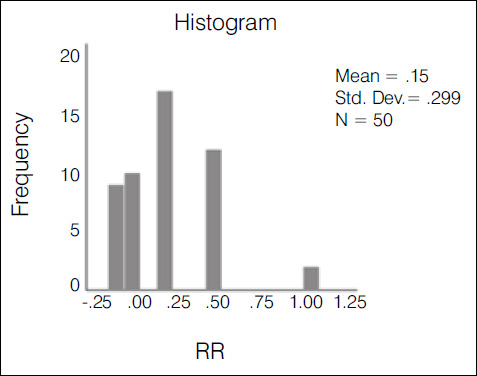
Histogram of the variation and concentration of kappa indices among R1 + R2

**Graph 4:**
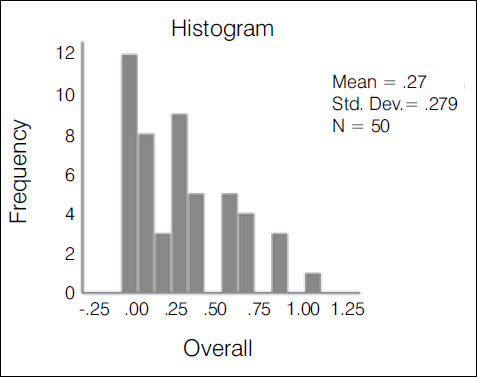
Histogram of the variation and concentration of kappa indices among all observers

The kappa agreement index was used in the statistical evaluation of the results. The evaluation of the 10 traumatologists had an index of 0.388-weak agreement (Graph 1); 3^rd^ year residents had an index of 0.235-weak agreement (Graph 2); 1st and second year residents had an index of 0.147-slight agreement (Graph 3); the overall result with all groups was 0.272-weak agreement (Graph 4).

**Graph 5:**
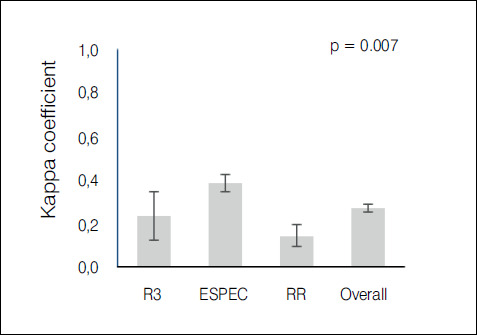
Graph presenting each group’s kappa coefficient with 95% CI, demonstrating that the traumatologists group had a statistically significant difference in relation to the other groups (p = 0.007)

It was demonstrated that the more experienced the group of evaluators, the higher the agreement index, with a statistically significant difference (p< 0,05) as is shown in Table 1. When comparing means and dispersions (with a 95% confidence interval) between the groups, we can see in Graph 5 that the dispersion among residents was also greater than among traumatologists.

**Table 1:** Comparison of the covering index using the ANOVA test with Bonferroni correction.

Group	Kappa	SD	95% CI
Tra	0.388	0.39	0.347 - 0.43
R3	0.235	0.57	0.122 - 0.348
R1 + R2	0.147	0.3	0.094 - 0.199
Overall	0.271	0.28	0.252 - 0.289

Kappa p-value: 0.7

Tra: Traumatologists

SD: Standard deviation

CI: Confidence index

## DISCUSSION

This study highlighted the low agreement among observers in the classification of trochanteric fractures divided into stable and unstable. The simple assessment of agreement made by organizing the number of cases in the same category has an intrinsic error - it cannot distinguish real agreement from random agreement. This subject has already been discussed by several authors who point out the need to incorporate random agreement in the evaluation to improve the reliability of the evaluation. ^(^ ^2^ ^),(^ ^3^

To correct for random agreement, in 1960 Cohen *et al* formulated the kappa coefficient. This measure evaluates the agreement between two observers and two objects adjusting for the possibility of random agreement. In 1971, Fleiss, based on the kappa coefficient, introduced the Fleiss kappa, which could evaluate agreement by correcting for random agreement between a larger number of fixed observers and more item classification options, as well as a greater number of items. ^(^ ^1^ ^),(^ ^4^ ^),(^ ^6^

This coefficient varies between -1 (complete disagreement) and +1 (complete agreement). There is no precise definition of an acceptable level of agreement. The variables between these denominators can be stratified more simply into: poor (< 5) and excellent (< 0.75). ^(^ ^5^ ^)^ According to Landis *et al*., another way to evaluate the result would be: poor (below 0), mild (0 - 0.2), weak (0.21 to 0.4), moderate (0.41 to 0.6), substantial (0.61 to 0.8) and almost perfect (0.81 to 1). ^(^ ^7^

The literature reveals several studies that aim to evaluate the reproducibility of diverse classifications related to orthopedics. Limiting the search to classifications related to trochanteric fractures and interobserver evaluation, we found the following results: Schwartzmann *et al* evaluated the AO classification and found a 0.34 kappa with subgroups and 0.6 without subgroups; Oliveira *et al* evaluated Tronzo’s rating and found a 0.44 kappa; Mattos *et al* evaluated the Tronzo and simplified AO classifications and found kappa of 0.36 and 0.53, respectively; Schipper *et al* evaluated the AO classification finding a 0.33 kappa with subgroups and 0.67 in the simplified one; Pervez *et al* evaluated Jansen’s classification by finding a 0.34 kappa; van Embeden *et al* evaluated Jansen and AO classifications by finding kappa of 0.48 and 0.4, respectively; Jim *et al* evaluated AO classifications with and without subgroups, Evans and Boyd by observers with more than 10 years of experience, finding kappa of 0.75, 0.41, 0.44 and 0.38, respectively; Yin *et al* evaluated the Evans, Jansen, AO, and Tang ratings and found kappa of 0.54, 0.53, 0.46, and 0.63, respectively. Cavaignac *et al* conducted a study using radiographies and tomographies with and without 3D reconstruction using Evans and AO classifications. This study found kappa of 0.28 and 0.50 in radiographies in AO and Evans classifications; AO simple tomography and Evans kappa of 0.33, 0.35, respectively; tomography with AO 3D reconstruction and Evans kappa of 0.28 and 0.47, respectively. ^(^ ^8^ ^)-(^ ^16^

The analysis of these studies reveals, at most, substantial results (0.61 - 0.8) in four articles when using the simplified AO classification in three groups and in one article using the Tang classification. We observed that more complex classifications, such as AO with its nine subgroups and Tronzo, always perform poorly (0.21 - 0.4). Evans and Jansen had a moderate agreement score (0.41 - 0.6). ^(^ ^8^ ^),(^ ^11^ ^),(^ ^12^ ^),(^ ^14^ ^),(^ ^15^

In this study, we proposed that the evaluators classify fractures in two ways: stable or unstable. Even with this extreme simplification, the kappa agreement index was 0.271, which is considered weak (0.21-0.4), when accounting for all observers. 

It is observed, as in other studies, that the observers’ level of professional experience influenced the result. ^(^ ^10^ When comparing the result obtained by isolating 1^st^ and 2^nd^ year residents (k = 0.147), 3^rd^ year residents (k = 0.235), and traumatologists (k = 0.388), a statistically significant difference was found. Although traumatologists reached a better agreement value, it is still very low, which makes this simple classification ineffective.

Considering that the use of classifications aims at discrimination, prognosis, and adequate treatment, we conclude that classifications with low reproducibility indexes that present high variability lose their clinical usefulness.

The December 1993 editorial of the Journal of Bone and Joint Surgery (JBJS) questioned the functionality and usefulness of the classifications. It presented the idea that classifications are tools that should always work in the same way and produce the same results. This tool should first prove to be functional and later prove to be useful in clinical practice. Considering these points, the JBJS is reluctant to accept studies that correlate classification with results. ^(^ ^17^

In light of this, we consider that classifications play an important role in clinical practice in orthopedics, but we must use them with the necessary level of criticism. It is evident from this study that the results analyzed in general are not reproducible with an acceptable level of agreement, even when making extreme simplifications. To be effective as work tools, kappa index classifications with rates lower than or equal to 0.8 should be improved or abandoned.

## CONCLUSION

This study showed a low interobserver agreement with a kappa of 0.272 in the classification of trochanteric fractures divided into STABLE and UNSTABLE. Based on this study, it is recommended that the classifications be extensively tested and reach a minimum level of interobserver reproducibility (kappa > 0.8). It is suggested that classifications that do not achieve this result be improved or abandoned.
